# Factors affecting the post-operative outcomes in patients aged over 80 following colorectal cancer surgery

**DOI:** 10.1007/s00384-022-04291-8

**Published:** 2023-01-12

**Authors:** Raymond Yap, Simon Wilkins, Mohammad Asghari-Jafarabadi, Karen Oliva, Wei Chun Wang, Suellyn Centauri, Paul J. McMurrick

**Affiliations:** 1 Cabrini Monash University Department of Surgery, Cabrini Hospital, Malvern, VIC 3144 Australia; 2 Department of Epidemiology and Preventive Medicine, School of Public Health and Preventive Medicine, Monash University, Melbourne, VIC 3004 Australia; 3 Cabrini Research, Cabrini Hospital, Malvern, VIC 3144 Australia; 4 School of Public Health and Preventive Medicine, Monash University, Melbourne, VIC 3004 Australia

**Keywords:** Colorectal cancer, Older patients, Patient outcomes, Surgical outcomes

## Abstract

**Purpose:**

In 2019, in Australia, there were 500,000 people aged 85 and over. Traditionally, clinicians have adopted the view that surgery is not desirable in this cohort due to increasing perioperative risk, perceived minimal clinical benefit, and shortened life expectancy. This cohort study is aimed at investigating postoperative outcomes from elective and non-elective colorectal cancer surgery in patients aged 80 and over.

**Methods:**

A retrospective analysis was conducted on patients from 2010 to 2020 on a prospectively maintained colorectal database. Patients aged over 80 who underwent surgical resection for colorectal cancer were reviewed. Oncological characteristics, short-term outcomes, overall survival, and relapse-free survival rates were analysed.

**Results:**

A total of 832 patients were identified from the database. Females comprised 55% of patients aged 80 and above. The median age was 84 for octogenarians and 92 for nonagenarians. Most patients were ASA 2 (212) or ASA 3 (501). ASA 3 and 4 and stage III pathology were associated with higher postoperative complications. Fifty percent of over 80 s and 37% of over 90 s were surgically discharged to their own home. Overall survival at 30, 180, and 360 days and 5 years was 98.1%, 93.1%, 87.2%, and 57.2% for the over 80 s and 98.1%, 88.9%, 74.9%, and 24.4% for the over 90 s.

**Conclusion:**

Our results demonstrate that surgical treatment of older patients is safe with acceptable short-, medium-, and long-term survival. Nonetheless, efforts are needed to reduce the rates of complications in older patients, including utilisation of multi-disciplinary teams to assess the optimal treatment strategy and postoperative care.

**Supplementary Information:**

The online version contains supplementary material available at 10.1007/s00384-022-04291-8.

## Introduction

In 2019, almost half a million of Australia’s population was aged 85 and over, while 1.2 million were aged 75 and over [1], with life expectancy continuing to grow [2]. The United Kingdom Department for Work and Pensions predicts that a girl born today has a one-in-three chance of living to 100, while a boy has a one-in-four chance [3]. Increasing age is a well-known risk factor for many types of cancer, including colorectal cancer [4]. Almost 40% of all new diagnoses of colorectal cancer in the USA are now in those patients aged 80 years or above, and approximately one-third of all colorectal cancer-related death is in this age group [5]. Older patients are increasingly contributing to a significant cohort of colorectal cancer patients worldwide; due to their age and consequent comorbidities, these patients present as complex treatment dilemmas. A survey of surgical oncologists in the USA and Europe found that those dealing with colorectal cancer were more likely to define “older patients” at a lower age and use a specific age cut-off not to offer elective cancer surgery [6].

Multiple reports have cited age as a risk factor for perioperative morbidity and mortality [7–9]. Traditionally, clinicians have frequently adopted the view that perioperative risks would be too high and future life expectancy too low in older patients and therefore offered less-aggressive oncological treatment [10]. In recent years, numerous reports have been published demonstrating that surgery in the over 80 s is feasible [11–13]. Additional studies have also shown 5-year survival data for the over 80 s ranging from 24% for colon cancer to 36–49% for rectal cancer [14–17]. However, 5-year survival data for the over 90 s is lacking for patients undergoing colorectal cancer surgery with only one study reporting 5-year survival of 20% and 34% for colon and rectal cancer respectively [14]. Little work has been done to stratify risks or the ability to select appropriate patients in these age groups to receive aggressive oncological treatment.

This study is aimed at investigating elective and non-elective colorectal cancer surgery outcomes in patients aged 80 years and above treated at both private and public hospitals in Melbourne, Australia, and at examining their short-, medium-, and long-term overall survival and disease-free survival.

## Materials and methods

A retrospective cohort study of older patients on the prospectively maintained colorectal neoplasia database, incorporating data from multiple sites, Melbourne, identified patients entered over a 10-year period between January 2010 and February 2020. Patients were included in this study according to selection criteria of age of 80 years old and above, diagnosed with colorectal cancer, and had undergone surgery for colorectal cancer. Data on patient demographics, perioperative risks, treatment, mortality and morbidity, and survival were collected. This database has demonstrated very high data completeness, accuracy, and patient follow-up [18]. If required and when available, additional data points were sourced from hospital databases and patient records. Patients were divided into two subgroups for statistical analysis, those aged 80–89 (octogenarian) at the time of surgery and those aged 90 and above (nonagenarian) at the time of surgery. Surgical entry was recorded at the time of surgery with patients categorised into three subgroups: open, minimally invasive techniques, and conversion from minimally invasive to open surgery. Urgent surgery was defined as surgery occurring within the same hospital admission, and emergency surgery was defined as surgery within the same day of hospital admission. A 30-day mortality was defined as death within 30 days following surgery. Anastomotic leak was defined as clinical or radiological evidence of a leak from the anastomosis. Relapse-free survival was determined as the length of time after the primary treatment for cancer ends that the patient survives without any clinical evidence of recurrence. Follow-up was left to individual surgeons or public clinic unit who adhered to the national (Cancer Council Australia) guidelines on follow-up after curative resection for colorectal cancer [19]. No patients were involved in the conduct of this study. Primary outcomes were postoperative outcomes and patient survival at 30, 90, 180, and 1-year post-surgery. Secondary outcomes were 5-year relapse-free survival and 5-year overall survival.

A clinical audit of routinely coded hospital data for preoperative admission location and postoperative disposition was performed. Patients were identified as presenting from three areas: home, another hospital or rehabilitation facility, or a supported care facility or aged care home. Discharge disposition was recorded similarly with a category for inpatient death as an outcome of the surgical admission.

### Statistical analyses

All analyses were conducted using R (version 4.2) [20]. Data were expressed using mean (SD) and median (percentile 25–percentile75) or (min–max) for numeric normal and non-normal variables, respectively, and frequency (percent) for categorical variables. For comparisons among groups defined by age categories, Fisher’s exact test and Mann–Whitney test were conducted, where appropriate. Logistic regression was used to analyse the association between clinical characteristics and perioperative complications, wherein odds ratios (ORs) and their 95% confidence interval (CI) were presented. To present the overall survival (OS) and relapse-free survival (RFS) probability of the patients, Kaplan–Meier curves were drawn. In addition, the median survival (95% CI), of the survival time was computed both by the age categories. Also, 1-, 3-, and 5-year survival probabilities and their 95% CI were also computed. The survival probabilities were compared across age categories using log-rank tests. Cox proportional hazard regressions were carried out for computing un-adjusted and adjusted hazard ratios (HRs), via univariable and multivariable modelling, respectively. Multivariable modelling was carried out either by enter strategy, wherein all variables from the univariable analyses were entered in the multivariable model. In addition, the proportional hazard (PH) assumption was assessed using Schoenfeld residual test for the final model. For multivariable modelling, the Harrell’s *c*-index was computed, which varies between 0 and 1, with 1 indicating the maximum agreement between observed and predicted deaths. Values greater than 0.7 indicate a fair agreement. The survival models’ coefficients were compared across age groups (80–89 vs. 90 + years) using Hausman specification test [21]. The confidence intervals were estimated using robust standard error to account for the clustering effect of the hospitals in the models. *p* values < 0.05 were considered as significant.

Ethics approval for this study was granted by the Cabrini Human Research Ethics Committee (Reference #02–30-04–18). All patients on the database gave informed consent. The research registry unique identifying number for this study is #7558 (www.researchregistry.com).

## Results

A total of 832 patients (858 treatment episodes) were identified as being 80 and over years of age at the time of diagnosis with colorectal cancer and undergoing colorectal cancer surgery from the colorectal neoplasia database within the study period. Males comprised 45% of patients ≥ 80 years of age. The median age at the time of diagnosis was 84 for the octogenarian (80–89 years) cohort and 92 for the nonagenarian cohort (over 90 years). Across both age groups, the majority of patients in the study were ASA 2 (212) or ASA 3 (501) at the time of surgery. The follow-up median was 17.88 months (0.07–60 months), with a 5-year cut-off. Supplemental Fig. 1 shows a flow chart of the number of patients in the study.

**Fig. 1:**
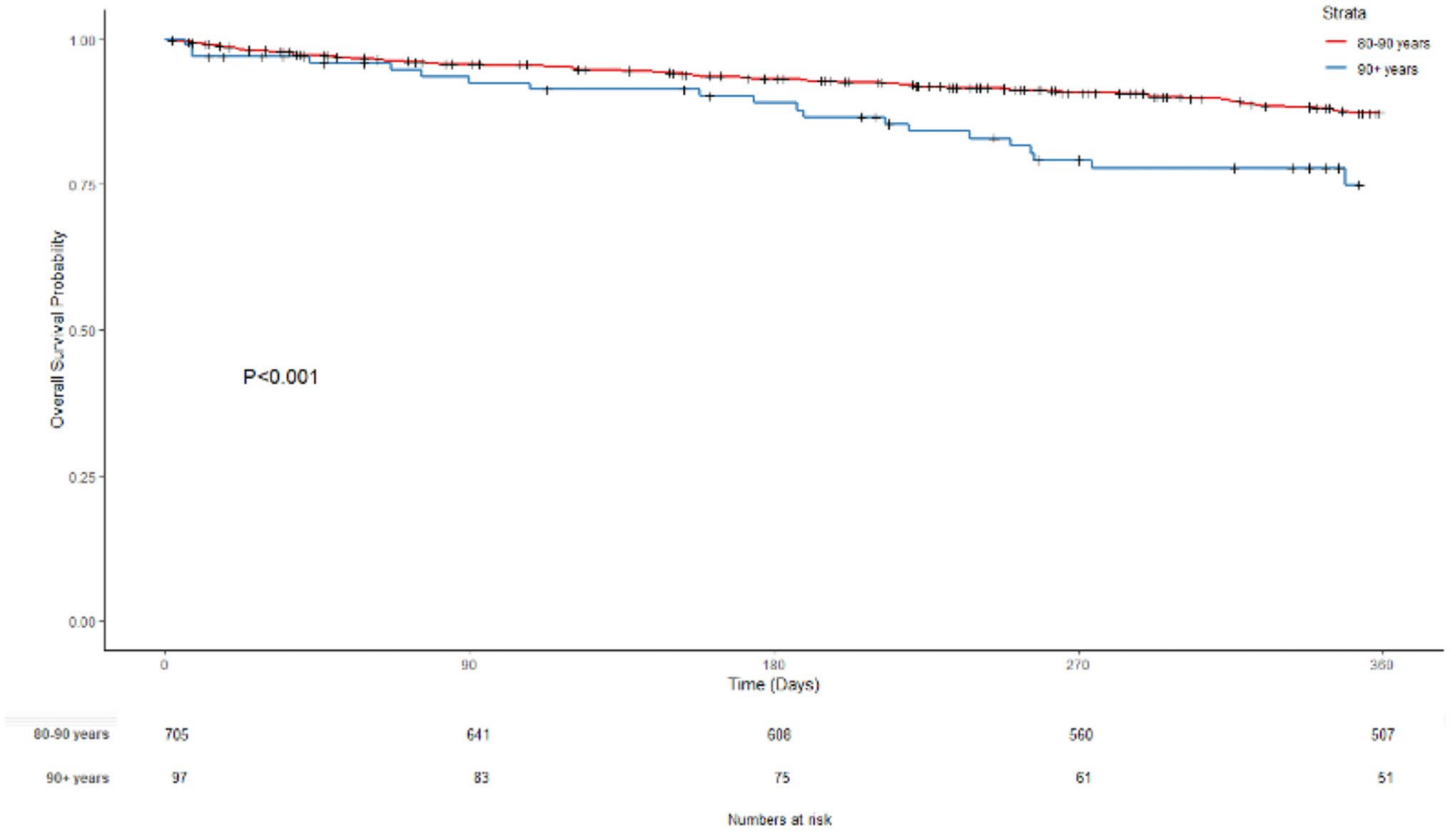
Overall survival probability

Table 1 describes the demographic details of the patients, divided into those in the ninth and tenth decades (and beyond) of life. When patient episodes were examined, there was an increasing comorbidity burden of patients in the older age group compared to the younger patients. The nonagenarian group had more patients who were of ASA 3 or 4 (89.8% vs. 67.1%, *p* < 0.001), more ischaemic heart disease (58.3% vs. 39.5%, *p* < 0.001), more patients receiving antiplatelet therapy (60.2% vs. 48.5%, *p* = 0.024), or more vasculopathy (55.6% vs. 41.1%, *p* < 0.005) than the octogenarian group. Nonagenarian patients were significantly more likely (*p* < 0.028) to have a BMI in a normal range (between 20 and 25).

**Table 1:** Demographic and comorbidity data of study patients

	Age 80–89	Age 90 +	*p* value
Patients (*n* = 832)	*n* = 726 (87.3%)	*n* = 106 (12.7)	
Age (median (range))	84 (80–89)	92 (90–101)	
Sex			0.815^F^
Male	330 (45.5)	47 (44.3)	
Female	396 (54.5)	59 (55.7)	
Patient episodes (*n* = 858)	*n* = 750 (87.4%)	*n* = 108 (12.6)	
BMI			**0.028**^F^
< 20	67 (9.5)	10 (10.8)	
20–25	293 (41.6)	52 (55.9)	
25–30	246 (34.9)	23 (24.7)	
30 +	98 (13.9)	8 (8.6)	
ASA			** < 0.001**^**F**^
1	16 (2.1)	0 (0.0)	
2	201 (30.8)	11 (10.2)	
3	432 (57.6)	69 (63.9)	
4	71 (9.5)	27 (25.9)	
PVD	78 (10.4)	11 (10.2)	> 0.999^F^
CVA	90 (12.0)	13 (12.0)	> 0.999^F^
IHD	296 (39.5)	63 (58.3)	** < 0.001**^**F**^
Vasculopathy	308 (41.1)	60 (55.6)	**0.005**^**F**^
Anti-platelet agent therapy	363 (48.5)	65 (60.2)	**0.024**^**F**^
Diabetes	138 (18.4)	15 (13.9)	0.284^F^
Respiratory disease	146 (19.5)	20 (18.5)	0.897^F^

*ASA* American Society of Anaesthesiologists Score, *BMI* body mass index, *CVA* cerebrovascular accident, *IHD* ischaemic heart disease, *PVD* peripheral vascular disease

*p* values for significant results are shown in bold

^F^Fisher’s exact test

When examining the types of surgical procedures between nonagenarians and octogenarians (Table 2), nonagenarians had significantly higher non-reconstructive left colonic surgery rates, with an almost tripling of the rate of Hartmann’s procedures (11.1% vs. 3.7%) though not significantly different. Unsurprisingly, this led to a corresponding reduction in the proportion of all types of anterior resection in the nonagenarian group. The incidence of stage I cancers in the over 90 s was lower than patient in their 80 s (17.3% vs. 30.0%, *p* = 0.031), whereas stage II (47.1% vs. 36.4%) and stage III (26.3% vs. 23.1%) were higher in the nonagenarian cohort (*p* = 0.031).

**Table 2:** Surgical characteristics and 30-day post-operative outcomes

	Age 80–89 *n* = 750 *n* (%)	Age 90 + *n* = 108 *n* (%)	*p* value
Procedure type			0.079^F^
Right hemicolectomy	321 (42.8)	49 (45.4)	
Extended right hemicolectomy	36 (4.8)	3 (2.8)	
Left hemicolectomy	31 (4.1)	5 (4.6)	
Sigmoid colectomy	8 (1.1)	1 (0.9)	
Total colectomy	7 (0.9)	1 (0.9)	
Sub-total colectomy	33 (4.4)	5 (4.6)	
Proctocolectomy	2 (0.3)	1 (0.9)	
High anterior resection	88 (11.7)	6 (5.6)	
Low anterior resection	61 (8.1)	8 (7.4)	
Ultra-low anterior resection	52 (6.9)	1 (0.9)	
APR	25 (3.3)	2 (1.9)	
Hartmann’s	28 (3.7)	12 (11.1)	
Miscellaneous	7 (0.9)	2 (1.9)	
Colo-anal anastomosis	1 (0.1)	0 (0.0)	
Transverse colectomy	16 (2.1)	4 (3.7)	
Local excision	1 (0.1)	0 (0.0)	
TEMS/TAMIS	15 (2.0)	4 (3.7)	
Laparotomy	321 (42.8)	49 (45.4)	
Other	36 (4.8)	3 (2.8)	
Urgency			0.062^F^
Emergency	34 (4.5)	6 (5.6)	
Urgent	72 (9.6)	18 (16.7)	
Elective	644 (85.9)	84 (77.8)	
Surgical entry			0.778^F^
Open	198 (26.4)	30 (27.8)	
Minimally invasive	437 (63.1)	65 (60.2)	
Conversion	76 (10.5)	13 (12.0)	
Overall stage^a^			**0.031**^**F**^
0 (pCR)	4 (0.6)	1 (0.9)	
I	223 (30.0)	19 (17.3)	
II	273 (36.4)	51 (47.1)	
III	174 (23.1)	29 (26.3)	
IV	74 (10.0)	9 (8.1)	
Surgical complications^b^	129 (18.5)	15 (14.0)	0.260^F^
Abdominal/pelvic collection	13 (1.9)	2 (1.9)	> 0.99^F^
Superficial wound dehiscence	3 (0.4)	1 (0.9)	0.434^F^
Deep wound dehiscence	5 (0.7)	0 (0.0)	> 0.99^F^
Wound infection	17 (2.4)	2 (1.9)	> 0.99^F^
Sepsis	9 (1.3)	1 (0.9)	> 0.99^F^
Prolonged ileus	59 (8.4)	5 (4.7)	0.180^F^
Ureteric injury	0 (0.0)	1 (0.9)	0.130^F^
Splenectomy	2 (0.3)	0 (0.0)	> 0.99^F^
Other surgical complications	27 (3.8)	0 (0)	**0.040**^**F**^
Medical complications^b^	127 (18.2)	13 (12.1)	0.120^F^
DVT/PE	5 (0.7)	0 (0.0)	> 0.99^F^
Chest infection	36 (5.1)	3 (2.8)	0.470^F^
Cardiac	40 (5.7)	4 (3.7)	0.500^F^
Other medical complications	71 (10.1)	7 (6.5)	0.240^F^
Any complication	239 (31.9)	28 (25.9)	0.224^F^
30-day mortality (*n* = 785)	14 (1.9)	2 (1.9)	> 0.999^F^
Anastomosis formed (*n* = 706)	654 (87.2)	83 (76.9)	**0.007**^**F**^
Anastomotic leak	16 (2.1)	2 (1.9)	> 0.999^F^
Stoma formed	158 (21.1)	24 (22.2)	0.912^F^
Organs resected	51 (6.8)	13 (12.0)	0.075^F^
Return to theatre	60 (8.0)	7 (6.5)	0.703^F^
Length of stay (median (IQR) (range))	10 (7–15) (1–74)	11 (8–16) (2–64)	0.123^ MW^
Neoadjuvant treatment	48 (6.4)	0 (0.0)	**0.003** ^F^
Adjuvant chemotherapy	75 (10.0)	1 (0.9)	** < 0.001**^**F**^
Adjuvant radiotherapy	0 (0.0)	3 (2.8)	**0.002** ^F^
Preoperative location			0.246^F^
Home	689 (91.9)	97 (89.8)	
Rehabilitation	31 (4.1)	3 (2.8)	
Supported care	30 (4.0)	8 (7.4)	
Postoperative location			**0.023**^**F**^
Home	375 (50.0)	40 (37.0)	
Rehabilitation	325 (43.3)	55 (50.9)	
Supported care	37 (4.9)	11 (10.2)	
Inpatient death	13 (1.7)	2 (1.9)	

^F^Fisher’s exact test; ^MW^Mann-Whitney test

*APR* abdominoperineal resection, *IQR* inter quartile range, *pCR* complete pathological response, *DVT* deep vein thrombosis, *PE* pulmonary embolism, *taTME* transanal total mesorectal excision, *TEMS* transanal endoscopic microsurgery, *TAMIS* transanal minimally invasive surgery

*p* values for significant results are shown in bold

^a^Patients with unknown staging excluded (5)

^b^Some patients had more than one complication

The overall surgical complication rate was 18.5% (octogenarian) vs. 14.0% (nonagenarian) (*p* = 0.26), while the medical complication rate was 18.2% vs 12.1% (*p* = 0.12) as described in Table 2. The types of surgical and medical complications did not differ between the two age groups. Less anastomoses were formed in the nonagenarian group (*p* = 0.007). A 30-day mortality was low for both groups (1.9%). There were no differences in the anastomotic leak rate between the two age groups (2.1% vs. 1.9%; *p* > 0.99) nor in the rate of patients returning to theatre, or length of stay of hospital admission. Neoadjuvant treatment, adjuvant chemotherapy, and/or adjuvant radiotherapy were rarely given to patients aged over 90 (Table 2).

Quality of life analysis demonstrates that octogenarians and nonagenarians came from similar levels of preoperative support, with the majority in each group admitted from home (91.9% and 89.8%, respectively, *p* = 0.246). Both groups suffered similar levels of inpatient death (1.7% and 1.9%). A much lower percentage of nonagenarians were discharged home (37.0% vs. 50.0%, *p* = 0.023), as most nonagenarians were referred for inpatient rehabilitation (50.9%). Octogenarians were less likely to be discharged to supported care accommodation (4.9% vs. 10.2%, *p* = 0.023; Table 2).

Univariable and multivariable analyses of factors affecting perioperative complications are shown in Table 3. In univariable analysis, ASA 3 (odds ratio (OR) 7.94, 95% confidence interval (CI) 1.59–144.24), ASA 4 (OR 9.47, 95% CI 1.80–174.97), and stage III disease (OR 2.19, 95% CI 1.43–3.36) were predictive factors for complications. Complications were reduced in patients having minimally invasive surgery (OR 0.66, 95% CI 0.47–0.93). In multivariable analyses, ASA 3 (OR 8.49, 95% CI 1.70–154.26), ASA 4 (OR 10.89, 95% CI 2.06–201.59), and stage III disease (OR 2.14, 95% CI 1.39–3.32) were predictive factors for perioperative complications in the study patient cohort. Patients over 90 years had a reduced risk of perioperative complications (OR 0.60, 95% CI 0.37–0.96).

**Table 3:** Univariable and multivariable analysis of factors affecting perioperative complications

Factor	Univariable analysis OR (95%CI)	Multivariable analysis OR (95%CI)
Patients (*n* = 832)		
Age		
80–89 yrs	Reference	Reference
90 + yrs	0.73 (0.45–1.15)	**0.60 (0.37–0.96)**
Episodes (*n* = 858)		
ASA		
1	Reference	Reference
2	4.39 (0.86–80.39)	4.49 (0.88–82.25)
3	**7.94 (1.59–144.24)**	**8.49 (1.70–154.26)**
4	**9.47 (1.80–174.97**	**10.89 (2.06–201.59)**
Urgency		
Elective	Reference	Reference
Emergency	0.78 (0.36–1.58)	0.64 (0.28–1.38)
Urgent	1.51 (0.96–2.37)	1.36 (0.83–2.21)
Surgical entry		
Open	Reference	Reference
Minimally invasive	**0.66 (0.47–0.93)**	0.71 (0.49–1.03)
Conversion	1.38 (0.84–2.28)	1.45 (0.86–2.45)
Overall stage		
0 (pCR)	1.87 (0.24–11.58)	1.74 (0.22–11.02)
I	Reference	Reference
II	1 (0.66–1.50)	0.93 (0.61–1.42)
III	**2.19 (1.43–3.36)**	**2.14 (1.39–3.32)**
IV	1.37 (0.78–2.40)	1.24 (0.67–2.26)

*ASA* American Society of Anaesthesiologists Score, *pCR* complete pathological response, *OR* odds ratio, *CI* confidence interval

*p* values for significant results are shown in bold

**Fig. 2:**
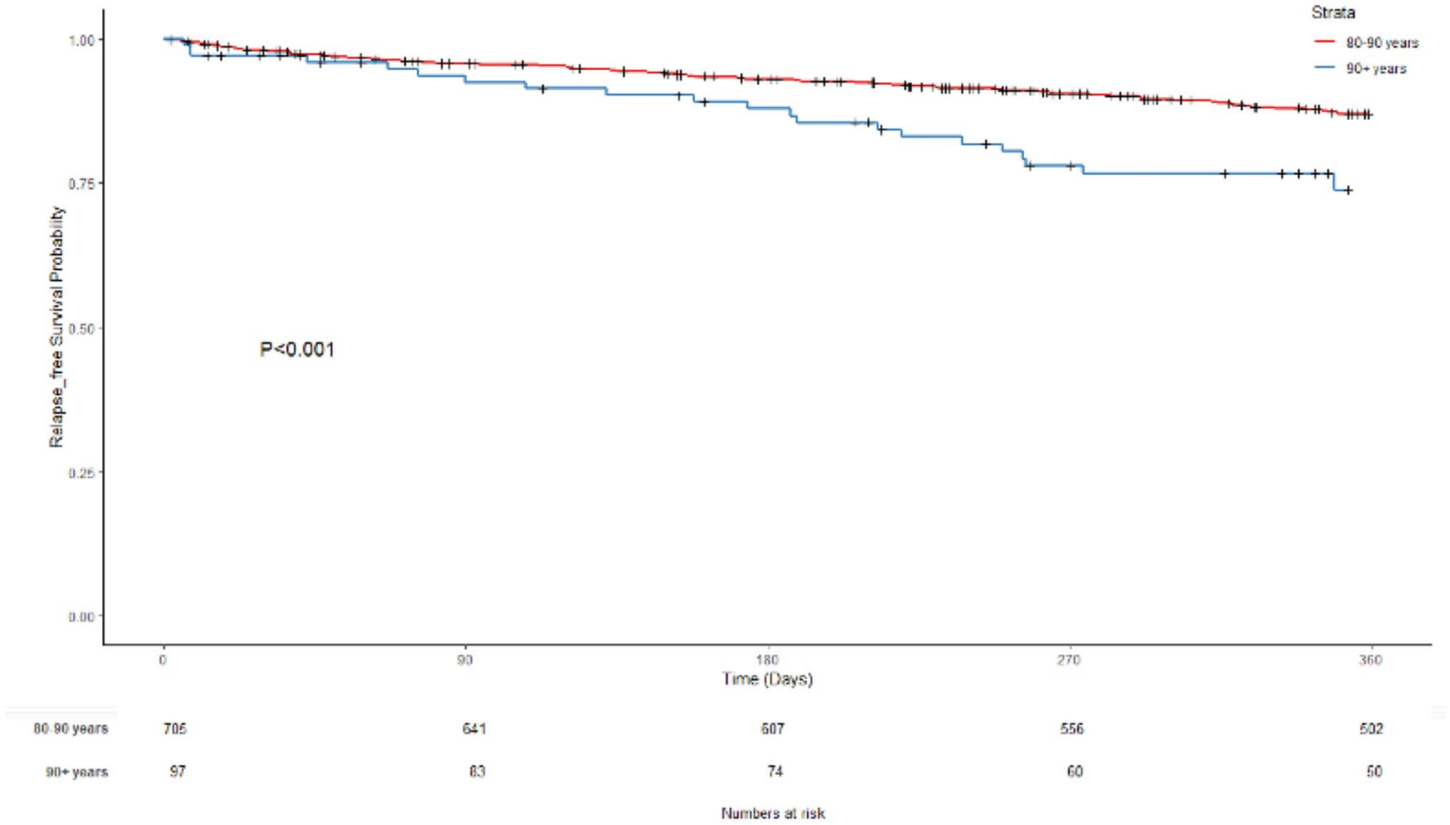
Relapse-free survival probability

The short-term, middle-term, and long-term overall survival of patients from both groups was also analysed. The survival of the octogenarians at 30 days, 90 days, 180 days, and 1 year was 98.1%, 95.7%, 93.1%, and 87.2%, respectively, whereas the survival of the nonagenarians at 30 days, 90 days, 180 days, and 1 year was 98.1%, 92.4%, 88.9%, and 74.9%, respectively. Higher overall survival was observed in patients aged 80–89 years compared with patients aged 90 + years. This result is reflected in a Kaplan–Meier curve for overall survival between the two groups (log rank test *p* < 0.001) (Fig. 1). Relapse-free survival over the same time periods demonstrated a similar pattern. The relapse-free survival of the octogenarians at 90 days, 180 days, and 1 year was 95.7%, 92.9%, and 86.9%, respectively. The relapse-free survival of the nonagenarians at 90 days, 180 days, and 1 year was 92.4%, 87.8%, and 73.7%, respectively. Higher relapse-free survival was observed in the 80–89 group (Fig. 2; log rank test *p* < 0.001).

The cohort was then analysed by age group to identify and compare differences in risk factors between groups using univariable and multivariable analyses. Table 4 shows the univariable analysis of factors affecting overall survival in the two groups. In the 80–89 group, several factors were associated with a significantly higher hazard to overall survival, such as BMI < 20 when compared to a BMI of 20–25, ASA scores of 3 or 4, urgent and emergency surgery, open surgery, any complications, formation of a stoma, and stage III or IV disease.

**Table 4:** Univariable analyses of factors affecting overall survival and their comparison across age categories

Variables	80–89 yrs		90 + yrs		Comparison *p* value^a^
	HR (95% CI)	*p* value	HR (95% CI)	*p* value	
Sex					
Male	Reference	–-	Reference	–-	
Female	0.87 (0.66–1.15)	0.319	1.06 (0.55–2.05)	0.867	0.515
BMI					
< 20	2.07 (1.32–3.23)	**0.001**	0.93 (0.30–2.88)	0.900	0.132
20–25	Reference	–-	Reference	–-	–-
25–30	0.81 (0.57–1.17)	0.265	1.35 (0.55–3.32)	0.507	0.224
30 +	1.03 (0.66–1.62)	0.888	0.63 (0.08–4.80)	0.654	0.623
ASA score					
2	Reference	–-	Reference	–-	–-
3	1.80 (1.27–2.56)	**0.001**	2.57 (0.60–11.06)	0.206	0.623
4	4.93 (3.10–7.83)	** < 0.001**	3.92 (0.86–17.84)	0.078	0.756
Urgency					
Emergency	3.25 (1.91–5.55)	** < 0.001**	9.91 (3.18–30.87)	** < 0.001**	**0.029**
Urgent	2.95 (2.02–4.31)	** < 0.001**	2.42 (1.07–5.46)	**0.034**	0.589
Elective	Reference	–-	Reference	–-	–-
Surgical entry					
Open	2.09 (1.54–2.84)	** < 0.001**	1.71 (0.82–3.58)	0.152	0.559
Minimally invasive	Reference	–-	Reference	–-	–-
Conversion	1.26 (0.79–2.01)	0.323	1.62 (0.66–3.96)	0.289	0.523
Surgical complications	1.92 (1.40–2.62)	** < 0.001**	1.87 (0.88–3.99)	0.106	0.940
Medical complications	1.73 (1.26–2.38)	**0.001**	1.65 (0.71–3.84)	0.242	0.912
Any complication	1.89 (1.43–2.51)	** < 0.001**	1.86 (0.95–3.63)	0.070	0.954
Stoma formed	1.51 (1.10–2.05)	**0.010**	1.47 (0.69–3.15)	0.317	0.952
Overall stage					
I	Reference	–-	Reference	–-	
II	1.32 (0.86–2.03)	0.201	5.10 (0.66–39.37)	0.118	0.185
III	2.28 (1.48–3.52)	** < 0.001**	12.11 (1.60–91.74)	**0.016**	0.098
IV	9.16 (5.86–14.31)	** < 0.001**	35.97 (4.31–299.88)	**0.001**	0.196
Adjuvant chemotherapy	1.26 (0.84–1.90)	0.263	3.12 (0.42–23.19)	0.267	0.367
Preoperative location					
Home	Reference	–-	Reference	–-	–-
Rehabilitation	1.07 (0.53–2.18)	0.848	NA	–-	1.000
Supported care	0.79 (0.33–1.93)	0.612	1.27 (0.39–4.18)	0.690	0.240
Postoperative location					
Home	Reference	–-	Reference	–-	–-
Rehabilitation	1.67 (1.24–2.26)	**0.001**	1.83 (0.83–4.00)	0.131	0.813
Supported care	1.13 (0.54–2.34)	0.745	4.34 (1.31–14.40)	**0.017**	**0.006**

*ASA* American Society of Anaesthesiologists Score, *BMI* body mass index, *NA* not applicable to be computed due to data sparsity, *HR* hazard ratio, *CI* confidence interval

*p* values for significant results are shown in bold

^a^Comparison in *p* value was estimated using Hausman specification test

Nonagenarians requiring emergency surgery were almost ten times more likely to have poor overall survival than those treated by elective surgery, whereas this risk was only doubled for those requiring urgent surgery. Octogenarian patients proceeding to rehabilitation after surgery were associated with a marginally increased risk (1.67) of poor overall survival compared to patients returning home. Nonagenarian patients discharged directly to supported care after surgery had almost 5 times higher risk of poor overall survival compared with patients returning home. In patients aged over 90, poor prognostic factors identified on univariate analysis were emergency and urgent surgery, stage III or IV colorectal cancer, these were associated with significantly a higher hazard to overall survival. When comparing the hazard ratios between the two groups, emergency surgery, stage III and IV, and patients returning to supported care hazard ratios were significantly higher in the 90 + group than the hazard ratios of the 80–89 group (Table 4). In multivariable analyses, a similar pattern was observed with emergency and urgent surgery, surgical and medical complications, and stage II and IV associated with higher hazard ratios for overall survival in the 80–89 group. These lost their significance in the 90 + group. Patients proceeding to rehabilitation, female patients, and those patients receiving adjuvant chemotherapy were associated with a significantly reduced hazard ratio for overall survival (Table 5). Comparison between groups demonstrated no significant differences in multivariable analysis hazard ratio.

**Table 5:** Multivariable analyses of factors affecting overall survival and their comparison across age categories

Variables	80–89 yrs		90 + yrs		Comparison *p* value^a^
	HR (95% CI)	*p* value	HR (95% CI)	*p* value	
Sex					
Male	Reference	–-	Reference	–-	–-
Female	0.70 (0.51–0.97)	**0.031**	0.66 (0.19–2.29)	0.515	0.926
BMI					
< 20	1.61 (0.97–2.66)	0.065	0.85 (0.20–3.58)	0.829	0.355
20–25	Reference	–-	Reference	–-	–-
25–30	0.92 (0.62–1.35)	0.669	1.18 (0.38–3.65)	0.778	0.649
30 +	0.91 (0.57–1.47)	0.700	2.37 (0.22–25.57)	0.477	0.421
ASA score					
2	Reference	–-	Reference	–-	–-
3	2.13 (0.51–8.86)	0.300	1.45 (0.13–15.90)	0.762	0.696
4	4.23 (0.95–18.80)	0.058	2.50 (0.12–50.98)	0.552	0.694
Urgency					
Emergency	2.08 (1.07–4.06)	**0.031**	0.66 (0.02–23.76)	0.819	0.522
Urgent	2.02 (1.28–3.18)	**0.003**	1.78 (0.39–8.18)	0.461	0.865
Elective	Reference	–-	Reference	–-	–-
Surgical entry					
Open	1.15 (0.77–1.72)	0.486	0.80 (0.22–2.88)	0.736	0.558
Minimally invasive	Reference	–-	Reference	–-	–-
Conversion	0.86 (0.51–1.45)	0.562	0.50 (0.07–3.55)	0.486	0.575
Surgical complications	1.79 (1.25–2.58)	**0.002**	1.47 (0.36–5.97)	0.588	0.776
Medical complications	1.47 (1.01–2.12)	**0.043**	1.50 (0.21–10.73)	0.685	0.980
Stoma formed	0.79 (0.53–1.16)	0.229	1.59 (0.47–5.41)	0.460	0.236
Overall stage					
I	Reference	–-	Reference	–-	–-
II	1.59 (0.98–2.58)	0.062	NA	–-	–-
III	2.80 (1.67–4.70)	** < 0.001**	NA	–-	–-
IV	11.70 (6.68–20.49)	** < 0.001**	NA	–-	–-
Adjuvant chemotherapy	0.55 (0.33–0.89)	**0.015**	1.25 (0.01–143.93)	0.926	0.730
Preoperative location					
Home	Reference	–-	Reference	–-	–-
Rehabilitation	0.59 (0.24–1.42)	0.238	NA	–-	–-
Supported care	0.80 (0.30–2.17)	0.662	0.68 (0.04–10.67)	0.781	0.898
Postoperative location					
Home	Reference	–-	Reference	–-	–-
Rehabilitation	1.42 (1.00–2.01)	**0.049**	2.06 (0.60–7.02)	0.249	0.548
Supported care	1.14 (0.46–2.81)	0.771	3.80 (0.42–34.08)	0.233	0.308

Harrell’s index for multivariable analysis (*C* = 0.779)

The proportional hazard assumption for all variables was satisfied using Schoenfeld residual test (all *p* > 0.05)

*ASA* American Society of Anaesthesiologists Score, *BMI* body mass index, *NA* not applicable to be computed due to data sparsity, *HR* hazard ratio, *CI* confidence interval

^a^Comparison in *p* value was estimated using Hausman specification test

*p* values for significant results are shown in bold

Relapse-free survival was associated with a number of poor prognostic factors for patients aged 80–89 (Table 6). A BMI of < 20, ASA 3 and 4, emergency and urgent surgery, open surgical procedures, surgical or medical complications, formation of a stoma, and stage III and IV were associated with a significant increase in hazard ratio for relapse-free survival (Table 6). Patients aged 80–89 who proceeded to rehabilitation after surgery also had an increased hazard ratio. In contrast, emergency surgery and urgent surgery, as well as stage III and IV, were associated with worse relapse-free survival in patients aged over 90 (Table 6). Nonagenarians who entered supportive care after surgery had worse relapse-free survival (Table 6). When comparing the two groups, emergency surgery and patients proceeding to supportive care following surgery showed significantly worse RFS outcomes in the 90 + group compared with the 80–89 group (Table 6). In multivariable analyses, a BMI < 20, ASA 4, emergency and urgent surgery, surgical complications, stage III and IV, and patients proceeding to rehabilitation after surgery were associated with worse outcomes for relapse-free survival (Table 7). Patients receiving adjuvant chemotherapy had a significantly improved hazard ratio improving their RFS (Table 7). There were no factors in the over 90 s that had a significant impact on RFS.

**Table 6:** Univariable analyses of factors affecting relapse-free survival and their comparison across age categories

Variables	80–89 yrs		90 + yrs		Comparison *p* value^a^
	HR (95% CI)	*p* value	HR (95% CI)	*p* value	
Sex					
Male	Reference	–-	Reference	–-	–-
Female	0.88 (0.67–1.17)	0.382	1.07 (0.55–2.07)	0.846	0.533
BMI					
< 20	2.08 (1.33–3.26)	**0.001**	0.91 (0.30–2.83)	0.877	0.120
20–25	Reference	–-	Reference	–-	–-
25–30	0.83 (0.58–1.19)	0.320	1.33 (0.54–3.25)	0.538	0.266
30 +	1.05 (0.67–1.64)	0.846	0.63 (0.08–4.79)	0.653	0.613
ASA score					
2	Reference	–-	Reference	–-	–-
3	1.78 (1.25–2.53)	**0.001**	2.56 (0.59–11.02)	0.207	0.618
4	5.09 (3.20–8.09)	** < 0.001**	3.95 (0.87–18.00)	0.076	0.731
Urgency					
Emergency	3.39 (1.98–5.79)	** < 0.001**	10.08 (3.24–31.41)	** < 0.001**	**0.033**
Urgent	2.93 (2.00–4.29)	** < 0.001**	2.50 (1.11–5.64)	**0.028**	0.663
Elective	Reference	–-	Reference	–-	–-
Surgical entry					
Open	2.15 (1.58–2.91)	** < 0.001**	1.70 (0.82–3.56)	0.156	0.501
Minimally invasive	Reference	–-	Reference	–-	–-
Conversion	1.26 (0.79–2.00)	0.338	1.61 (0.66–3.94)	0.296	0.522
Surgical complications	1.71 (1.24–2.35)	**0.001**	1.65 (0.71–3.84)	0.242	0.934
Medical complications	1.71 (1.24–2.35)	**0.001**	1.65 (0.71–3.84)	0.242	0.934
Any complication	1.87 (1.41–2.48)	** < 0.001**	1.86 (0.95–3.64)	0.069	0.992
Stoma formed	1.49 (1.09–2.03)	**0.012**	1.45 (0.68–3.09)	0.340	0.937
Overall stage					
I	Reference	–-	Reference	–-	–-
II	1.34 (0.88–2.06)	0.175	5.11 (0.66–39.40)	0.118	0.190
III	2.30 (1.49–3.55)	** < 0.001**	12.27 (1.62–92.95)	**0.015**	0.097
IV	9.19 (5.88–14.36)	** < 0.001**	34.98 (4.20–291.55)	**0.001**	0.206
Adjuvant chemotherapy	1.24 (0.82–1.87)	0.302	3.17 (0.43–23.58)	0.259	0.349
Preoperative location					
Home	Reference	–-	Reference	–-	–-
Rehabilitation	1.06 (0.52–2.16)	0.865	0.00 (0.00)	1.000	1.000
Supported care	0.79 (0.32–1.92)	0.605	1.26 (0.38–4.13)	0.705	0.247
Postoperative location					
Home	Reference	–-	Reference	–-	–-
Rehabilitation	1.70 (1.26–2.29)	**0.001**	1.84 (0.84–4.03)	0.127	0.829
Supported care	1.13 (0.54–2.34)	0.745	4.30 (1.29–14.27)	**0.017**	**0.006**

*ASA* American Society of Anaesthesiologists Score, *BMI* body mass index, *NA* not applicable to be computed due to data sparsity, *HR* hazard ratio, *CI* confidence interval

^a^Comparison in *p* value was estimated using Hausman specification test

*p* values for significant results are shown in bold

**Table 7:** Multivariable analyses of factors affecting relapse-free survival and their comparison across age categories

Variables	80–89 yrs		90 + yrs		Comparison *p* value^a^
	HR (95% CI)	*p* value	HR (95% CI)	*p* value	
Sex					
Male	Reference	–-	Reference	–-	–-
Female	0.73 (0.53–1.01)	0.056	0.74 (0.21–2.59)	0.639	0.980
BMI					
< 20	1.68 (1.02–2.77)	**0.043**	0.76 (0.18–3.25)	0.716	0.256
20–25	Reference	–-	Reference	–-	–-
25–30	0.95 (0.64–1.40)	0.792	1.06 (0.34–3.35)	0.914	0.834
30 +	0.97 (0.60–1.56)	0.896	2.36 (0.22–25.27)	0.479	0.453
ASA score					
2	Reference	–-	Reference	–-	–-
3	1.35 (0.91–2.00)	0.142	1.62 (0.15–18.01)	0.693	0.877
4	2.95 (1.66–5.24)	** < 0.001**	2.86 (0.14–59.53)	0.498	0.983
Urgency					
Emergency	2.18 (1.11–4.29)	**0.024**	0.65 (0.02–24.37)	0.817	0.507
Urgent	1.99 (1.26–3.15)	**0.003**	1.85 (0.41–8.40)	0.427	0.920
Elective	Reference	–-	Reference	–-	–-
Surgical entry					
Open	Reference	–-	Reference	–-	–-
Minimally invasive	0.79 (0.54–1.18)	0.252	1.29 (0.37–4.53)	0.691	0.427
Conversion	0.70 (0.40–1.21)	0.202	0.62 (0.10–4.00)	0.616	0.895
Surgical complications	1.76 (1.22–2.52)	**0.002**	1.51 (0.37–6.13)	0.566	0.825
Medical complications	1.44 (0.99–2.08)	0.054	1.28 (0.17–9.74)	0.809	0.911
Stoma formed	0.75 (0.51–1.12)	0.157	1.47 (0.44–4.91)	0.535	0.253
Overall stage					
I	Reference	–-	Reference	–-	–-
II	1.60 (0.99–2.61)	0.057	NA	–-	–-
III	2.95 (1.76–4.96)	** < 0.001**	NA	–-	–-
IV	12.02 (6.83–21.16)	** < 0.001**	NA	–-	–-
Adjuvant chemotherapy	0.52 (0.32–0.85)	**0.009**	1.48 (0.01–181.15)	0.874	0.670
Preoperative location					
Home	Reference	–-	Reference	–-	–-
Rehabilitation	0.56 (0.23–1.35)	0.197	NA	–-	–-
Supported care	0.78 (0.29–2.11)	0.632	0.72 (0.05–10.78)	0.810	0.944
Postoperative location					
Home	Reference	–-	Reference	–-	–-
Rehabilitation	1.43 (1.01–2.02)	**0.043**	2.21 (0.64–7.71)	0.212	0.475
Supported care	1.13 (0.46–2.76)	0.794	4.15 (0.45–38.21)	0.209	0.209

Harrell’s index for multivariable analysis (*C* = 0.766)

The proportional hazard assumption for all variables were satisfied using Schoenfeld residual test (all *p* > 0.05)

*ASA* American Society of Anaesthesiologists Score, *BMI* body mass index, *NA* not applicable to be computed due to data sparsity, *HR* hazard ratio, *CI* confidence interval

*p* values for significant results are shown in bold

^a^Comparison in *p* value was estimated using Hausman specification test

Supplementary Fig. 2 shows a Kaplan–Meier curve for overall survival in the two patient groups. Five-year overall survival was 57.2% in the octogenarian group and 24.4% in the nonagenarian group (log rank test *p* < 0.000134). Relapse-free survival was 57.0% and 24.6% for the octogenarian and nonagenarian groups, respectively (Supplementary Fig. 3; log rank test *p* = 0.000149). Overall survival and relapse-free survival over 1, 3, and 5 years are summarised in Supplementary Table 1.

## Discussion

An average 90-year-old in Australia has a less than 15% chance of making it to their 95th birthday, while an 80-year-old patient has a 70% chance of making it to their 85^th^ birthday [22]. In this study, short-term survival was only significantly different between the two age groups at one year (87.2% vs. 74.9%). At 180 days, the two groups were not significantly different (93.1% vs. 88.9%), with a mortality rate of 11.1%, similar to our previously published 10.4% mortality rate in a smaller cohort of patients over 90 [23]. In a Spanish study of 74 patients, postoperative mortality was 25% [24], much higher than our 30-day mortality of 1.9% and 180-day and 360-day mortality of the over 90 s of 88.9% and 74.9%, respectively. In contrast, a Japanese study demonstrated 0% mortality within 180 days of surgery for 48 patients that had undergone laparoscopic surgery [25].

Predominantly, the demographic and comorbidity data were similar between the two different age groups in this cohort. The over 90 s group tended to have additional comorbidities such as known heart disease or vasculopathy with a corresponding higher ASA but had lower BMIs, perhaps reflecting a better overall health status in those patients who come to surgery and survive to that age. The traditionally used ASA score for perioperative risk stratification was higher in the nonagenarians. The type of surgical procedure and the surgical urgency were the same in the two groups. However, the nonagenarian cohort had slightly more open or conversion procedures and more Hartmann’s procedures and was more likely to have an advanced pathological cancer stage. This more advanced presentation is likely due to the reduced screening of patients in their octogenarian years as per national guidelines [19], leading to later presentations of malignancy. There was no difference in the rate or type of surgical complications between the two groups nor the 30-day mortality, identical at 1.9%. The low rate of 30-day mortality compares favourably with a previously published rate for colorectal cancer patients aged over 80 of 9.3% [26].

The results in this study support the evidence that operations on elderly patients with colorectal cancer result in reduced overall survival compared to younger patients. Previous studies, including our own, showed that patients who do receive surgery have a reasonable quality of life and the ability to return to their own homes [23, 27]. Increasing age decile from octogenarian to nonagenarian showed some impact on postoperative disposition. Half of the octogenarians and 37% of the nonagenarians receiving surgery for colorectal cancer in this cohort returned to their pre-operative level of independent living at home on discharge. A study of long-term functional decline after colorectal surgery reported that the odds of functional decline increased by 2.08 per decade of age over 65 [28]. Consequently, it was an expected finding that over 50% of nonagenarians in this study were discharged from acute inpatient care to an inpatient rehabilitation facility. In Australia, rehabilitation services are provided to carefully selected patients who need assistance with improving physical and mental endurance to manage the sequelae of medical and surgical treatments. It is the aim that patients offered inpatient rehabilitation return to their former independent status. A recent German study of colorectal cancer patients has also suggested an association of inpatient rehabilitation with better overall and disease-specific survival [29].

Our study showed that overall survival for patients aged 80–89 was adversely affected by urgent or emergency surgery, stage III or IV pathology, and surgical or medical complications. Factors that led to increased postoperative complications included ASA 3 or 4 and stage III cancers. In this study, patients aged over 90 had less risk of postoperative complications. This contrasts with a previous study where age greater than 75 was independently associated with postoperative complications [30]. Our study shows that in the 80–89 patient cohort, the presence of any postoperative complication had an extremely adverse effect on the patient’s overall prognosis. The result is that any measures to minimise or eliminate postoperative complications in this population should be investigated, even if they may have a marginal immediate benefit. Previous studies have demonstrated several non-modifiable factors that can lead to complications, including our own works showing that patients with a higher BMI or diabetic patients are more prone to complications after surgery [31, 32]. However, surgical techniques can be modified to reduce the risk of complications such as an anastomotic leak, for example, by ensuring any anastomosis is tension-free or by using intra-operative endoscopy to assess rectal anastomoses [33]. Our retrospective study demonstrated that oversewing of stapled anastomoses at the time of surgery reduced the incidence of anastomotic leaks [34]; admittedly, this remains controversial as a German Delphi study reported that there was no consensus to routinely oversew anastomoses [33]. In this study, only surgical complications influenced relapse-free survival in the over 80–89 group.

The short-term survival for patients following colorectal cancer surgery was good. The 1-year survival rate was 87.2% for the octogenarians and 74.9% for the nonagenarians. Relapse-free survival after 1 year was also similar, with 86.9% for the 80–89 group and 73.7% for the 90 + group. In terms of long-term survival, our results show that 5-year overall survival for carefully selected patients undergoing surgery for colorectal cancer for both octogenarian and nonagenarian groups to be acceptable. The 5-year overall survival rate for the 80–89 group was 57.2%, and 24.4% for the nonagenarian group. These rates are higher than previously reported studies where patients aged over 80 without comorbidities had a 39% 5-year OS rate for colon cancer and 32.9% for rectal cancer [15], or where patients aged 80–84 had an OS rate of 49%, and patients aged over 85 had a rate of 36% [16]. Similar figures were described in a French study where colon cancer patients aged 80–84 had an OS of 40%, those aged 85–89 had a rate of 44% [14]. In the same study, colon cancer patients over 90 had an OS rate of 20.5% [14]. The reason behind the 4% increase in 5-year overall survival in this nonagenarian cohort is likely due to selection bias in the study design. Previous studies reporting overall survival rates for octogenarians and nonagenarians did not distinguish between untreated and treated colorectal cancer survival rates. This study’s overall post-surgery survival rates contrast strongly with the reported 5-year overall survival rate of only 4.4% for those patients with untreated rectal cancer [35]. Australian life expectancy has increased dramatically in recent decades. The average 80-year-old in Australia has a life expectancy of around 10 years, while a 90-year-old can be expected to live another 4.5 years [22]. However, traditional colorectal cancer screening programs generally stop well before the age of 80, with 74 years of age being the cut-off in Australia. Given that benefits from colorectal cancer screening are only seen after 5–10 years, this study may add some impetus to consider colorectal screening in a more elderly population if their life expectancy is likely to be greater than 5 years. In our analysis, the fact that emergency surgery and later-stage cancers contributed to poor patient outcomes is no surprise. Greater risk of poor outcomes was three times higher in the nonagenarians than octogenarians (*p* = 0.029). In younger patient cohorts (ages 50–74), colorectal cancer mortality is reduced by 36% by screening [36]. Although life expectancy is shorter in an older cohort, our data suggests that preventing late-stage cancer and emergency presentations may have a similar effect on perioperative outcomes.

This study has the usual limitations of being a retrospective analysis of patients; however, this is mitigated somewhat by the prospective data entry in the colorectal neoplasia database used. This study is not a large-scale multicentre study and the number of patients included is not sufficient to draw a definitive conclusion as to whether patients with colorectal cancer in their 80 s or 90 s should or should not undergo surgery. Additionally, as mentioned earlier, this study only captured patients in the age group that were deemed fit for surgical treatment and no comment on the survival of those not offered surgery can be made.

## Conclusions

This study demonstrates that surgical treatment of select patients in the later years of life is safe and should be strongly considered within a multidisciplinary format before surgery. Utmost preparation is needed to reduce the rates of both postoperative complications and emergency surgery in this patient cohort, who suffer more in consequence than younger patients in terms of their long-term prognosis. Care should be taken when counselling older patients for operative management as quality of life and post-operative outcomes must be considered. The next step in this arena is to look at specific interventions to improve the quality of care provided to this population and then measure the effect on the quality of life in these patients.

### Supplementary Information

Below is the link to the electronic supplementary material.

Supplementary file1 (PDF 37 KB)

Supplementary file2 (PDF 11 KB)

## Data Availability

Study participants were assured raw data would remain confidential and not be shared.

## Declarations

### Disclaimer

The funder had no role in the conduct of this study nor in the preparation of this manuscript.
