# Supplementary material for: Factors affecting the post-operative outcomes in patients aged over 80 following colorectal cancer surgery
Source: Int J Colorectal Dis. 2023 Jan 12;38(1):11. doi: 10.1007/s00384-022-04291-8 (PMC9836984; doi:10.1007/s00384-022-04291-8)

Factors affecting the post-operative outcomes in patients aged over 80 following colorectal cancer surgery

Yap et al

Supplemental Figure S1:

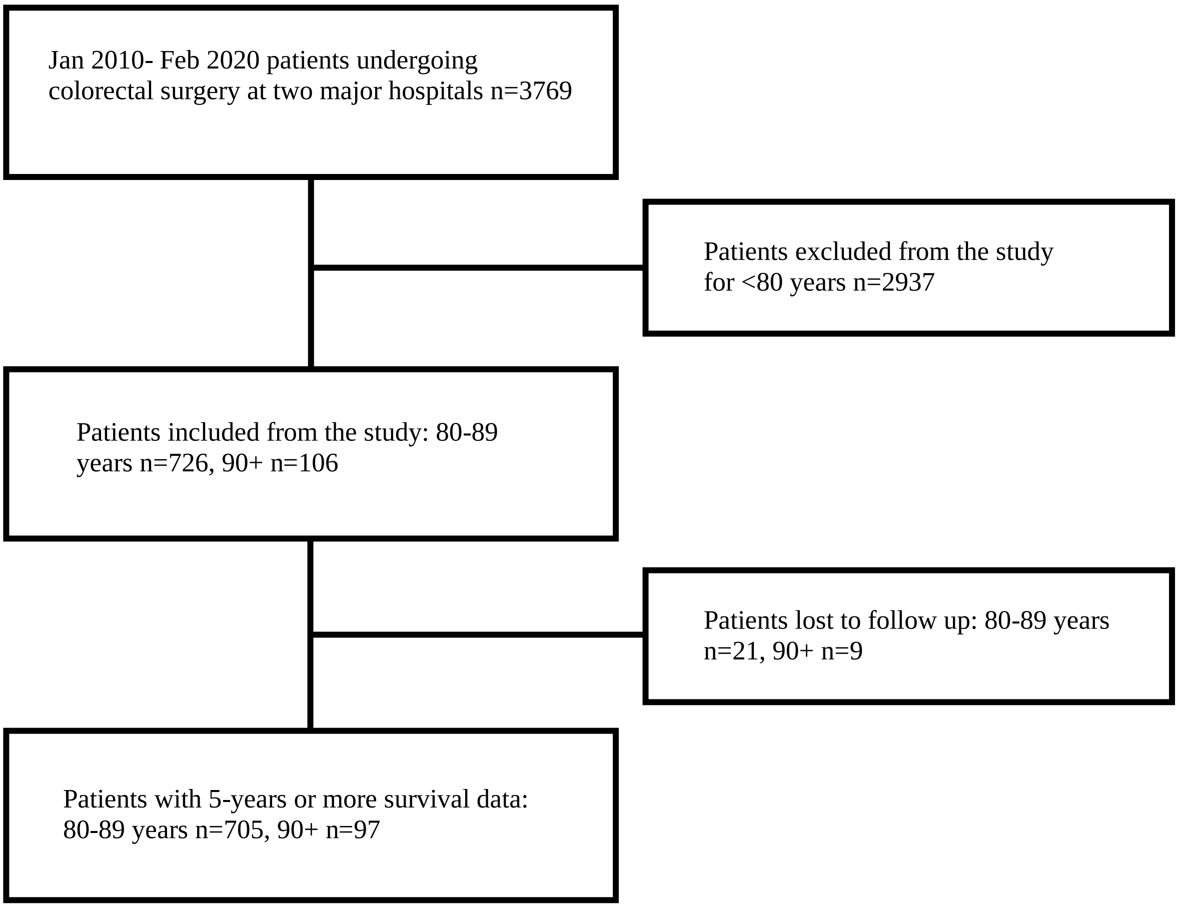

Supplemental Figure S2: Kaplan-Maier curve for overall survival across 80-90 and 90+ age categories

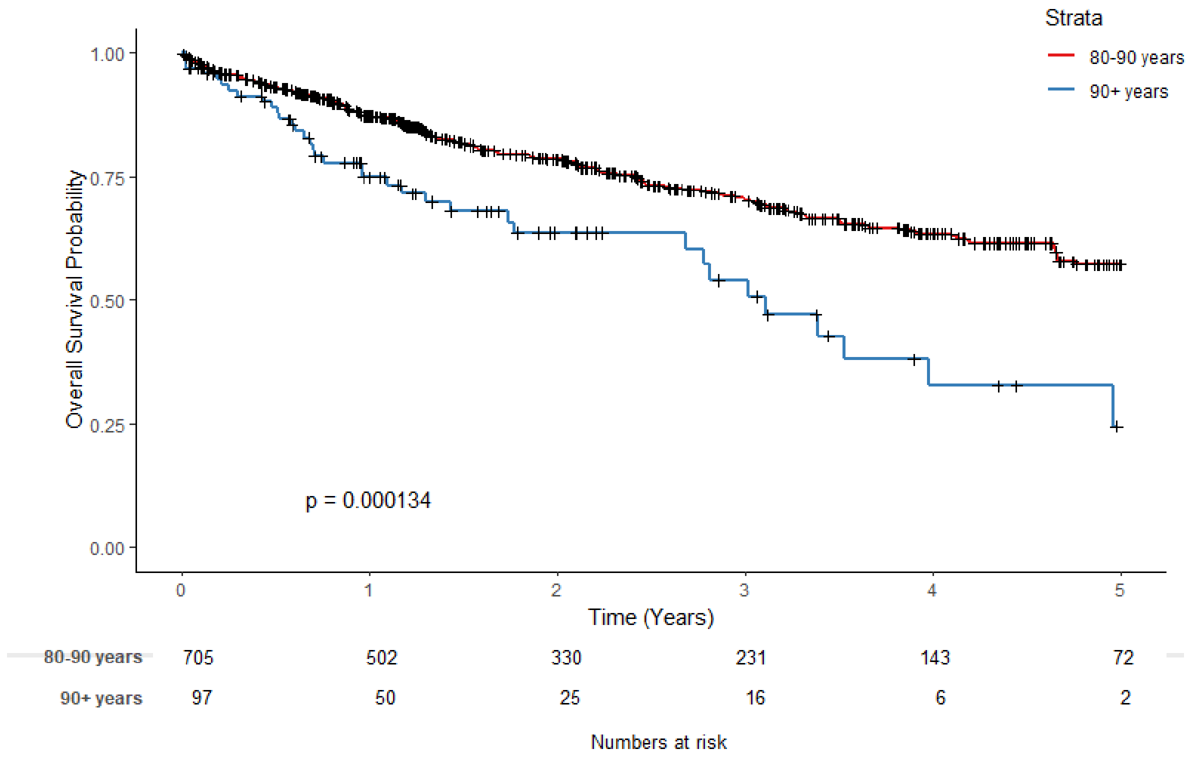

Supplemental Figure S3: Kaplan-Maier curve for relapse-free survival across 80-90 and 90+ age categories

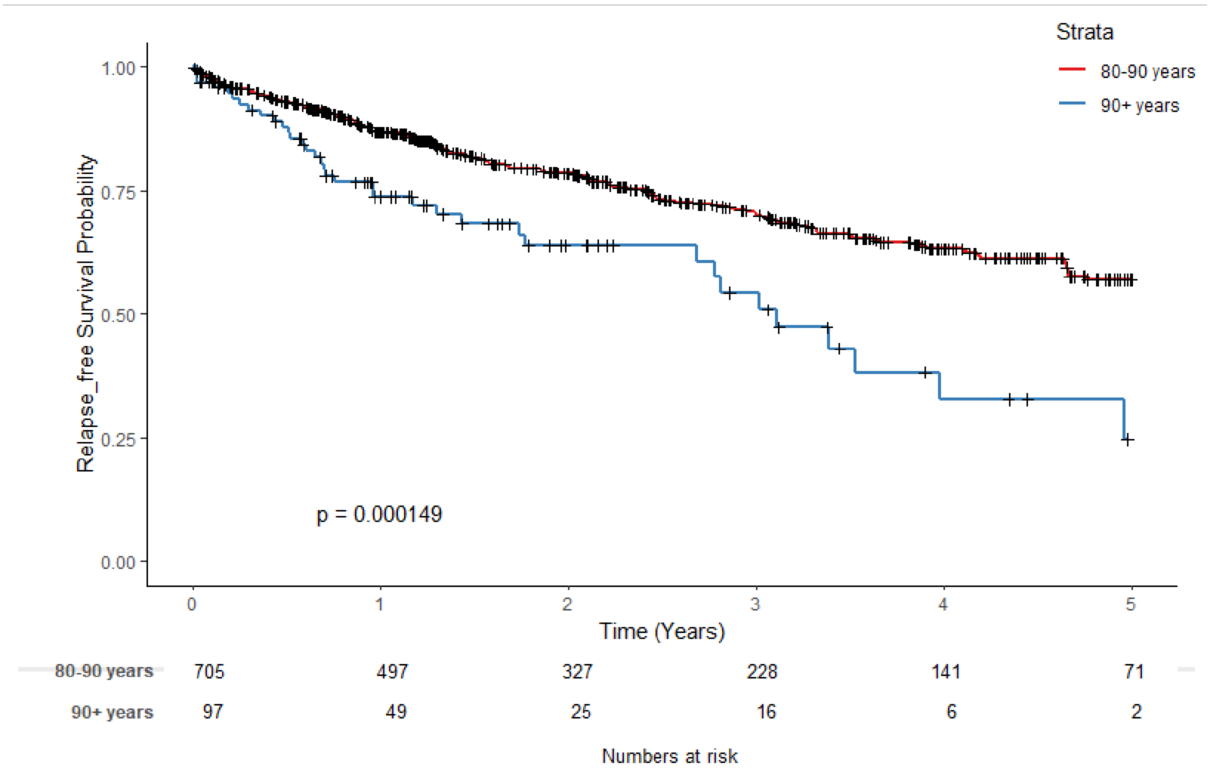

Supplement: Supplementary file 1 — Supplementary file1 (PDF 37 KB) [file 384_2022_4291_MOESM1_ESM.pdf]
