# Supplementary material for: Factors affecting the post-operative outcomes in patients aged over 80 following colorectal cancer surgery
Source: Int J Colorectal Dis. 2023 Jan 12;38(1):11. doi: 10.1007/s00384-022-04291-8 (PMC9836984; doi:10.1007/s00384-022-04291-8)
Supplement: Supplementary file 2 — Supplementary file2 (PDF 11 KB) [file 384_2022_4291_MOESM2_ESM.pdf]

Factors affecting the post-operative outcomes in patients aged over 80 following colorectal cancer surgery

Yap et al

Supplementary Table S1: Survival measures of patients across age categories

	**Overall survival**		**Relapse free survival**	
**Survival Measures**	**80-90 years**	**90+ years**	**80-90 years**	**90+ years**
**1-year survival (95% CI)**	0.872 (0.847-0.898)	0.749 (0.661-0.849)	0.869 (0.843-0.895)	0.737 (0.649-0.839)
**3-year survival (95% CI)**	0.701 (0.661-0.744)	0.540 (0.417-0.700)	0.700 (0.660-0.743)	0.543 (0.420-0.703)
**5-year survival (95% CI)**	0.572 (0.519-0.630)	0.244 (0.112-0.532)	0.570 (0.517-0.628)	0.246 (0.113-0.534)

CI: Confidence Interval
